# Supplementary material for: Patient satisfaction after outpatient hysteroscopy: a retrospective descriptive study
Source: PeerJ. 2025 Nov 10;13:e20272. doi: 10.7717/peerj.20272 (PMC12614103; doi:10.7717/peerj.20272)
Supplement: Supplemental Information 1 [file peerj-13-20272-s001.docx]

**CUESTIONARIO DE SATISFACCIÓN EN HISTEROSCOPIA AMBULATORIA**

1. Información recibida previa histeroscopia

- Suficiente
- Intermedia
- Insuficiente
- No recuerda

2. ¿Volvería a realizar el procedimiento a nivel ambulatorio?

- Sí
- No
- No lo tiene claro

3. ¿Recomendaría el procedimiento?

- Sí
- No

4. Grado de satisfacción general del procedimiento

- Puntuación 0-10
- Si ≤ 5: Explicar el motivo.

5. ¿Remisión de la clínica o motivo por el cual se realizó la histeroscopia?

- Completamente
- Remisión parcial
- No remisión
- Empeoramiento
- No valorable (asintomática, procedimiento únicamente diagnóstico...)

***English translation***

**SATISFACTION QUESTIONNAIRE IN OUTPATIENT HYSTEROSCOPY**

1. Information received prior to hysteroscopy

- Sufficient
- Intermediate
- Insufficient
- Doesn’t recall

2. Would you undergo the procedure in office again?

- Yes
- No
- Doesn’t know

3. Would you recommend the procedure?

- Yes
- No

4. Overall satisfaction level

- Score 0-10
- If ≤ 5: Explain the reason

5. Remission of symptoms or reason for performing the hysteroscopy?

- Total remission
- Partial remission
- No remission
- Worsening
- Not assessable (asymptomatic, only diagnostic procedure...)
