# Supplementary material for: Patient satisfaction after outpatient hysteroscopy: a retrospective descriptive study
Source: PeerJ. 2025 Nov 10;13:e20272. doi: 10.7717/peerj.20272 (PMC12614103; doi:10.7717/peerj.20272)
Supplement: Supplemental Information 2 [file peerj-13-20272-s002.docx]

|  | | Satisfaction grade | Age | Hysteroscopist | Hysteroscopy Dx/Tx | Hysteroscope used | Indication | Symptoms | Ecographic diagnosis | Hysteroscopy diagnosis | Pathology | Procedure | Successful HSC | OR referral | Menopausal status | Nº vaginal deliveries | C-Section | Previous info | Anxiety/Depression | Outpatient HSC again? | Change to OR | Recommend | Symptom remission |
| --- | --- | --- | --- | --- | --- | --- | --- | --- | --- | --- | --- | --- | --- | --- | --- | --- | --- | --- | --- | --- | --- | --- | --- |
| Pearson correlation | Satisfaction grade | 1,000 | -,039 | -,084 | -,017 | ,059 | ,062 | ,082 | ,014 | -,169 | -,117 | ,011 | -,177 | ,150 | ,036 | -,021 | ,150 | -,421 | ,134 | -,395 | ,397 | -,621 | ,009 |
|  | Age | -,039 | 1,000 | -,064 | ,105 | -,011 | -,238 | -,397 | -,080 | -,056 | -,118 | -,075 | ,070 | ,069 | -,689 | ,162 | ,048 | ,013 | -,075 | ,038 | ,125 | -,024 | ,117 |
|  | Hysteroscopist | -,084 | -,064 | 1,000 | -,061 | -,080 | ,058 | ,117 | -,099 | -,011 | ,080 | -,016 | -,031 | ,035 | ,020 | -,013 | -,058 | ,048 | -,034 | ,048 | -,028 | ,042 | -,128 |
|  | Hysteroscopy Dx/Tx | -,017 | ,105 | -,061 | 1,000 | ,337 | -,171 | -,070 | ,042 | ,065 | -,292 | ,582 | -,146 | ,134 | -,066 | -,044 | ,066 | -,094 | ,025 | ,039 | ,010 | ,048 | -,010 |
|  | Hysteroscope used | ,059 | -,011 | -,080 | ,337 | 1,000 | -,218 | -,072 | ,166 | ,142 | -,075 | ,259 | -,026 | ,005 | ,026 | -,063 | ,033 | -,163 | ,029 | -,012 | -,004 | ,011 | ,090 |
|  | Indication | ,062 | -,238 | ,058 | -,171 | -,218 | 1,000 | ,471 | -,266 | -,102 | ,253 | -,090 | ,065 | ,002 | ,292 | ,053 | -,003 | ,002 | ,049 | -,081 | ,069 | -,096 | -,050 |
|  | Symptoms | ,082 | -,397 | ,117 | -,070 | -,072 | ,471 | 1,000 | ,018 | ,101 | ,358 | ,099 | -,057 | -,013 | ,303 | -,023 | ,006 | ,022 | ,028 | -,047 | -,055 | -,053 | -,269 |
|  | Ecographic diagnosis | ,014 | -,080 | -,099 | ,042 | ,166 | -,266 | ,018 | 1,000 | ,363 | ,030 | ,176 | ,036 | -,118 | -,039 | ,075 | -,091 | -,036 | ,065 | -,067 | -,035 | -,054 | ,062 |
|  | Hysteroscopy diagnosis | -,169 | -,056 | -,011 | ,065 | ,142 | -,102 | ,101 | ,363 | 1,000 | ,319 | ,217 | ,556 | -,408 | ,025 | -,006 | -,030 | ,061 | -,071 | ,087 | -,102 | ,145 | ,021 |
|  | Pathology | -,117 | -,118 | ,080 | -,292 | -,075 | ,253 | ,358 | ,030 | ,319 | 1,000 | -,242 | ,317 | -,257 | ,008 | ,052 | -,118 | ,131 | -,022 | ,050 | -,128 | ,072 | -,020 |
|  | Procedure | ,011 | -,075 | -,016 | ,582 | ,259 | -,090 | ,099 | ,176 | ,217 | -,242 | 1,000 | -,170 | ,182 | ,122 | -,035 | ,067 | -,071 | ,057 | -,045 | ,030 | ,005 | -,036 |
|  | Successful HSC | -,177 | ,070 | -,031 | -,146 | -,026 | ,065 | -,057 | ,036 | ,556 | ,317 | -,170 | 1,000 | -,596 | -,083 | ,083 | ,012 | ,132 | -,099 | ,149 | -,123 | ,142 | ,051 |
|  | OR referral | ,150 | ,069 | ,035 | ,134 | ,005 | ,002 | -,013 | -,118 | -,408 | -,257 | ,182 | -,596 | 1,000 | ,003 | -,004 | -,046 | -,087 | ,112 | -,106 | ,163 | -,115 | ,016 |
|  | Menopausal status | ,036 | -,689 | ,020 | -,066 | ,026 | ,292 | ,303 | -,039 | ,025 | ,008 | ,122 | -,083 | ,003 | 1,000 | -,087 | ,066 | -,023 | ,110 | -,135 | ,000 | -,038 | -,039 |
|  | Nº vaginal deliveries |  | ,162 | -,013 | -,044 | -,063 | ,053 | -,023 | ,075 | -,006 | ,052 | -,035 | ,083 | -,004 | -,087 | 1,000 | ,011 | -,017 | ,007 | ,001 | -,021 | -,046 | -,063 |
|  | C-Section | ,150 | ,048 | -,058 | ,066 | ,033 | -,003 | ,006 | -,091 | -,030 | -,118 | ,067 | ,012 | -,046 | ,066 | ,011 | 1,000 | -,139 | ,020 | -,116 | ,068 | -,081 | ,042 |
|  | Previous info | -,421 | ,013 | ,048 | -,094 | -,163 | ,002 | ,022 | -,036 | ,061 | ,131 | -,071 | ,132 | -,087 | -,023 | -,017 | -,139 | 1,000 | -,117 | ,165 | -,147 | ,324 | -,078 |
|  | Anxiety/ Depression | ,134 | -,075 | -,034 | ,025 | ,029 | ,049 | ,028 | ,065 | -,071 | -,022 | ,057 | -,099 | ,112 | ,110 | ,007 | ,020 | -,117 | 1,000 | -,149 | ,106 | -,171 | ,113 |
|  | Outpatient HSC again? | -,395 | ,038 | ,048 | ,039 | -,012 | -,081 | -,047 | -,067 | ,087 | ,050 | -,045 | ,149 | -,106 | -,135 | ,001 | -,116 | ,165 | -,149 | 1,000 | -,448 | ,605 | -,004 |
|  | Change to OR | ,397 | ,125 | -,028 | ,010 | -,004 | ,069 | -,055 | -,035 | -,102 | -,128 | ,030 | -,123 | ,163 | ,000 | -,021 | ,068 | -,147 | ,106 | -,448 | 1,000 | -,504 | ,084 |
|  | Recommend | -,621 | -,024 | ,042 | ,048 | ,011 | -,096 | -,053 | -,054 | ,145 | ,072 | ,005 | ,142 | -,115 | -,038 | -,046 | -,081 | ,324 | -,171 | ,605 | -,504 | 1,000 | -,118 |
|  | Symptom remission | ,009 | ,117 | -,128 | -,010 | ,090 | -,050 | -,269 | ,062 | ,021 | -,020 | -,036 | ,051 | ,016 | -,039 | -,063 | ,042 | -,078 | ,113 | -,004 | ,084 | -,118 | 1,000 |
| Sig. (unilateral) | Satisfaction grade | . | ,231 | ,059 | ,377 | ,133 | ,121 | ,061 | ,395 | ,001 | ,014 | ,422 | ,000 | ,002 | ,249 | ,350 | ,002 | ,000 | ,006 | ,000 | ,000 | ,000 | ,430 |
|  | Age | ,231 | . | ,117 | ,025 | ,420 | ,000 | ,000 | ,066 | ,147 | ,013 | ,081 | ,094 | ,096 | ,000 | ,001 | ,183 | ,405 | ,080 | ,237 | ,010 | ,329 | ,014 |
|  | Hysteroscopist | ,059 | ,117 | . | ,128 | ,067 | ,138 | ,014 | ,031 | ,415 | ,067 | ,380 | ,280 | ,256 | ,352 | ,406 | ,137 | ,182 | ,261 | ,185 | ,302 | ,218 | ,008 |
|  | Hysteroscopy Dx/Tx | ,377 | ,025 | ,128 | . | ,000 | ,001 | ,095 | ,216 | ,110 | ,000 | ,000 | ,003 | ,006 | ,110 | ,203 | ,108 | ,038 | ,319 | ,234 | ,426 | ,186 | ,426 |
|  | Hysteroscope used | ,133 | ,420 | ,067 | ,000 | . | ,000 | ,088 | ,001 | ,004 | ,079 | ,000 | ,310 | ,463 | ,312 | ,119 | ,268 | ,001 | ,291 | ,409 | ,472 | ,417 | ,045 |
|  | Indication | ,121 | ,000 | ,138 | ,001 | ,000 | . | ,000 | ,000 | ,028 | ,000 | ,046 | ,111 | ,489 | ,000 | ,162 | ,476 | ,486 | ,181 | ,066 | ,099 | ,035 | ,175 |
|  | Symptoms | ,061 | ,000 | ,014 | ,095 | ,088 | ,000 | . | ,366 | ,029 | ,000 | ,032 | ,141 | ,407 | ,000 | ,336 | ,454 | ,339 | ,301 | ,188 | ,150 | ,159 | ,000 |
|  | Ecographic diagnosis | ,395 | ,066 | ,031 | ,216 | ,001 | ,000 | ,366 | . | ,000 | ,286 | ,000 | ,249 | ,013 | ,233 | ,080 | ,044 | ,253 | ,111 | ,104 | ,256 | ,154 | ,122 |
|  | Hysteroscopy diagnosis | ,001 | ,147 | ,415 | ,110 | ,004 | ,028 | ,029 | ,000 | . | ,000 | ,000 | ,000 | ,000 | ,320 | ,455 | ,287 | ,127 | ,092 | ,052 | ,028 | ,003 | ,345 |
|  | Pathology | ,014 | ,013 | ,067 | ,000 | ,079 | ,000 | ,000 | ,286 | ,000 | . | ,000 | ,000 | ,000 | ,442 | ,166 | ,013 | ,007 | ,338 | ,175 | ,008 | ,088 | ,357 |
|  | Procedure | ,422 | ,081 | ,380 | ,000 | ,000 | ,046 | ,032 | ,000 | ,000 | ,000 | . | ,001 | ,000 | ,011 | ,256 | ,105 | ,091 | ,143 | ,199 | ,285 | ,464 | ,251 |
|  | Successful HSC | ,000 | ,094 | ,280 | ,003 | ,310 | ,111 | ,141 | ,249 | ,000 | ,000 | ,001 | . | ,000 | ,061 | ,060 | ,409 | ,007 | ,032 | ,003 | ,011 | ,004 | ,169 |
|  | OR referral | ,002 | ,096 | ,256 | ,006 | ,463 | ,489 | ,407 | ,013 | ,000 | ,000 | ,000 | ,000 | . | ,481 | ,469 | ,193 | ,052 | ,018 | ,024 | ,001 | ,015 | ,384 |
|  | Menopausal status | ,249 | ,000 | ,352 | ,110 | ,312 | ,000 | ,000 | ,233 | ,320 | ,442 | ,011 | ,061 | ,481 | . | ,051 | ,108 | ,334 | ,020 | ,005 | ,499 | ,238 | ,232 |
|  | Nº vaginal deliveries | ,350 | ,001 | ,406 | ,203 | ,119 | ,162 | ,336 | ,080 | ,455 | ,166 | ,256 | ,060 | ,469 | ,051 | . | ,420 | ,373 | ,448 | ,489 | ,346 | ,197 | ,118 |
|  | C-Section | ,002 | ,183 | ,137 | ,108 | ,268 | ,476 | ,454 | ,044 | ,287 | ,013 | ,105 | ,409 | ,193 | ,108 | ,420 | . | ,005 | ,353 | ,015 | ,100 | ,064 | ,217 |
|  | Previous info | ,000 | ,405 | ,182 | ,038 | ,001 | ,486 | ,339 | ,253 | ,127 | ,007 | ,091 | ,007 | ,052 | ,334 | ,373 | ,005 | . | ,014 | ,001 | ,003 | ,000 | ,071 |
|  | Anxiety/ Depression | ,006 | ,080 | ,261 | ,319 | ,291 | ,181 | ,301 | ,111 | ,092 | ,338 | ,143 | ,032 | ,018 | ,020 | ,448 | ,353 | ,014 | . | ,003 | ,023 | ,001 | ,017 |
|  | Outpatient HSC again? | ,000 | ,237 | ,185 | ,234 | ,409 | ,066 | ,188 | ,104 | ,052 | ,175 | ,199 | ,003 | ,024 | ,005 | ,489 | ,015 | ,001 | ,003 | . | ,000 | ,000 | ,468 |
|  | Change to OR | ,000 | ,010 | ,302 | ,426 | ,472 | ,099 | ,150 | ,256 | ,028 | ,008 | ,285 | ,011 | ,001 | ,499 | ,346 | ,100 | ,003 | ,023 | ,000 | . | ,000 | ,058 |
|  | Recommend | ,000 | ,329 | ,218 | ,186 | ,417 | ,035 | ,159 | ,154 | ,003 | ,088 | ,464 | ,004 | ,015 | ,238 | ,197 | ,064 | ,000 | ,001 | ,000 | ,000 | . | ,013 |
|  | Symptom remission | ,430 | ,014 | ,008 | ,426 | ,045 | ,175 | ,000 | ,122 | ,345 | ,357 | ,251 | ,169 | ,384 | ,232 | ,118 | ,217 | ,071 | ,017 | ,468 | ,058 | ,013 | . |

**Supplementary Table 1**. Multiple linear regression table.
